# Preparation of Degradable and Transformable Core–Corona-Type Particles that Control Cellular Uptake by Thermal Shape Change

**DOI:** 10.1021/acsbiomaterials.3c01554

**Published:** 2024-01-20

**Authors:** Syuuhei Komatsu, Satoshi Yamada, Akihiko Kikuchi

**Affiliations:** Department of Materials Science and Technology, Tokyo University of Science, 6-3-1 Niijuku, Katsushika, Tokyo 125-8585, Japan

**Keywords:** 2-methylene-1, 3-dioxepane, degradable particles, thermal shape change, cellular uptake

## Abstract

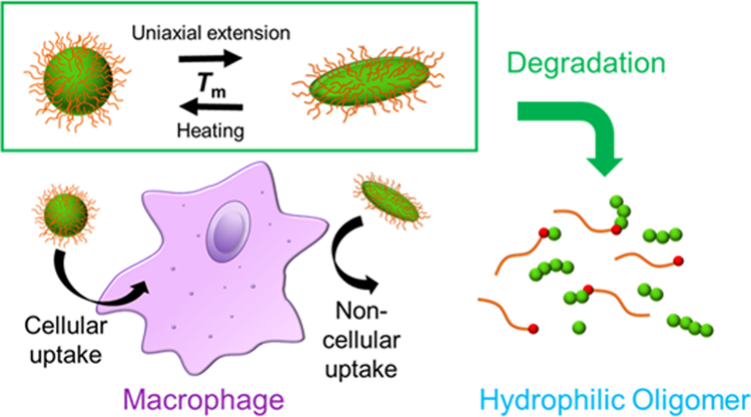

Particle–cell interactions, such as cellular uptake, vary depending on the particle size, shape, and surface properties. By dynamic control of the physical properties of particles, microparticle–cell interactions can intentionally be altered. Particle degradability is also necessary for their application in the body. In this study, we aimed to prepare degradable core–corona-type particles that are deformed near the body temperature and investigated particle shape-dependent cellular uptake. Degradable and transformable particles consisting of poly(2-methylene-1,3-dioxepane)-*co*-poly(ethylene glycol) with three-armed poly(ε-caprolactone) (PCL) were prepared. The particle melting point was controlled by the chain length of the three-armed PCL. Particle degradation occurred under both acidic and alkaline conditions via ester group hydrolysis in the polymer backbones. The rod-shaped microparticles prepared by uniaxial stretching at a temperature above the melting point of the core showed less uptake into macrophages than did the spherical microparticles. Therefore, the degradable transformable particles enable macrophage interaction control via stimuli-regulated particle shapes and are expected to be applied as drug delivery carriers that can be decomposed and excreted from the body.

## Introduction

Polymeric nano- and microparticles have been applied in a variety of fields, especially in biomedical applications such as drug delivery,^1−3^ imaging,^4,5^ and diagnostic carriers,^6,7^ because of their large surface areas and simple surface functionalization.^8−11^ Various types of polymeric particles have been developed using variety types of materials with stimuli-responsive and biodegradable properties.^12,13^ In addition, controlling the interactions between particles and cells, such as endocytosis and phagocytosis, is a key issue for drug delivery system (DDS) carriers. The particle size^14^ and shape^15^ affect cellular uptake. Therefore, the interaction between the particles and cells can be controlled by dynamically adjusting physical properties. Mitragotri et al. reported cellular uptake suppression of rod-shaped particles by macrophages and dendritic cells.^16−19^ Therefore, particle design that escapes or promotes phagocytosis is vital for DDS carriers. We reported the preparation of transformable particles from rod shape to spherical shape above the glass transition temperature (*T*_g_) of the particle core.^19^ The *T*_g_ of the particle core can be controlled by controlling the polymer composition of the particle core, which consists of methyl methacrylate and butyl methacrylate. Therefore, the particle shapes, rods or spheres, can be controlled at appropriate temperatures using the composition of the core materials.

Biodegradable properties are often required for the particles used in living bodies. Biodegradable particles based on poly(ε-caprolactone) (PCL),^20,21^ poly(d,l-lactide-*co*-glycolide) (PLGA),^22^ polypeptides,^23^ and polysaccharides^24^ have attracted interest as they release drugs while undergoing degradation and are then excreted from the body. Recently, cyclic ketene acetal monomers have attracted attention for the design of biomaterials because they can introduce ester groups in the polymer backbone via conventional radical polymerization. These monomers include 2-methylene-1,3-dioxepane^25−29^ and 5,6-benzo-2-methylene-1,3-dioxepane.^30−32^ Previously, we reported biodegradable core–corona-type nanoparticles with a poly(ethylene glycol) (PEG) corona prepared by a precipitation method of poly(2-methylene-1,3-dioxepane)-*ran*-poly(ethylene glycol) (PMDO-*co*-PEG).^33,34^ The PMDO-*co*-PEG nanoparticles containing the antitumor drug doxorubicin (DOX) were absorbed by HeLa cells, and decreased cell viability was observed due to doxorubicin (DOX) release in cells via acidic degradation. While degradable materials have a variety of properties, on the other hand, design of materials with degradability and transformable properties is difficult.

**Figure 1:**
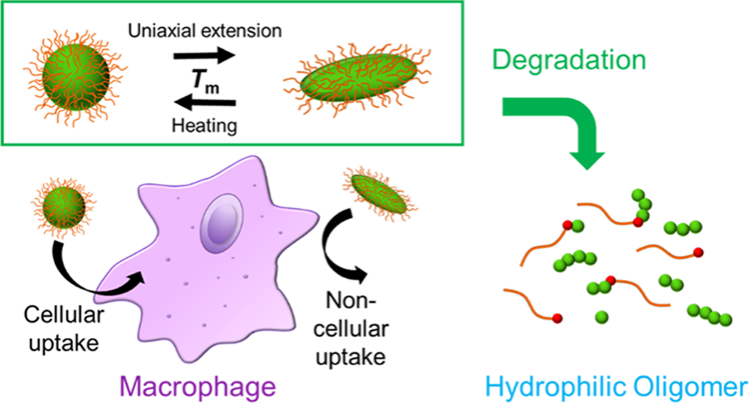
Schematic illustration of the biodegradable and transformable particles, representing controlled cellular uptake using particle shapes.

In this study, we focused on degradable particles composed of PMDO-*co*-PEG and PCL with controlled melting temperatures. Linear PCL has a melting point of approximately 60 °C;^35^ thus, linear PCL cannot be applied to shape-changing materials via melting temperature in the living body (with a basal temperature of 37 °C). In contrast, the melting point of PCL is controlled by its branched structure and chain length.^36^ Therefore, degradable and transformable particles were prepared by using PMDO-*co*-PEG and branched PCL with melting points near the body temperature. In addition, a high number of particles of a few micrometers were phagocytosed by macrophages.^37^ Therefore, mixing PCL allows simultaneous control of the melting point and particle size. Herein, we report the preparation of transformable and degradable core–corona-type particles for controlled cellular interactions using thermally controlled physical properties (Figure 1). Three-armed PCL (3PCL-5) was prepared by ring-opening polymerization to control the melting temperature. The diameters of the prepared particles were altered by varying the feed ratio of PMDO-*co*-PEG and 3PCL-5. In addition, the ester groups in PMDO-*co*-PEG and 3PCL-5 were hydrolyzed to form water-soluble, low-molecular weight compounds. The rod-shaped particles were prepared by uniaxial stretching of the particles within the poly(vinyl alcohol) (PVA) film above the melting point of 3PCL-5. At 37 °C, near the melting point, the rod-shaped particles rapidly returned to spherical particles. Finally, rod-shaped and spherical particles were suspended in cultured RAW 264.7 macrophage cells to assess the effect of the different shapes on phagocytosis.

## Experimental Section

### Materials

Chloroacetaldehyde, dimethylacetal, and poly(ethylene glycol) monomethacrylate (PEG) (nominal molecular weight of 2000 Da) were purchased from Sigma-Aldrich (MO). 1,4-Butanediol, potassium *t*-butoxide (*t*-BuOK), 1,4-dioxane, tetrahydrofuran (THF), Dowex 50 (H^+^), dimethyl sulfoxide (DMSO), 2,2′-azobis(isobutyronitrile) (AIBN), phosphate buffer saline (PBS), poly(vinyl alcohol) (PVA) (indicates the degree of polymerization), diethyl ether, penicillin–streptomycin solution, 0.5% (w/v) trypsin-5.3 mmol/L EDTA·4Na solution (10-fold concentration), methanol, 6-hexanolactone (ε-caprolactone), 1,1,1-tris(hydroxymethyl)propane (trimethylolpropane), tin(II) 2-ethylhexanoate, and formalin were purchased from Fujifilm Wako Pure Chemical Corporation (Osaka, Japan). Fetal bovine serum (FBS) was purchased from Funakoshi Co. (Tokyo, Japan). Nile Red was purchased from Tokyo Chemical Industry (Tokyo, Japan). DMSO was purified by distillation under reduced pressure, and the fraction boiling at 95.0 °C (0.5 kPa) was collected and used. Dulbecco’s modified Eagle medium (DMEM) powder with high glucose and pyruvate (10 × 1 L) and the LIVE/DEAD viability/cytotoxicity kit for mammalian cells were purchased from Thermo Fisher Scientific Co. (Waltham, MA). 2-Methylene-1,3-dioxepane (MDO) was synthesized by a two-step reaction according to a previous report.^33^ PMDO-*co*-PEGs were then synthesized by radical copolymerization of MDO and PEG according to a previous report.^33^

### Three-Armed PCL Synthesis

A predetermined amount of trimethylolpropane (TMP), which was used as an initiator for the ring-opening polymerization of e-caprolactone, was added to a round-bottom flask and dried overnight at 25 °C under reduced pressure. ε-Caprolactone (CL) (50 mmol, 5.54 mL) was added under a dry nitrogen atmosphere and mixed until the TMP was completely dissolved in the CL. Tin(II) 2-ethylhexanoate (Sn(Oct)_2_) (200 μL) was added under a nitrogen atmosphere. After vacuum nitrogen replacement was performed five times, the inside of the flask was filled with dry nitrogen gas, and a N_2_ gas-filled balloon was attached to the top of the three-way stopcock. The mixture was heated to 120 °C in an oil bath and polymerized for 24 h. The obtained sample was dissolved in 20 mL of THF and dropwise added to 150 mL of a mixed solution of hexane:diethyl ether of 1:1 (v/v) for reprecipitation. The mixture was allowed to stand for 1 h to precipitate the polymer sample. The supernatant containing unreacted substances was removed, and the polymer sample was recovered by vacuum filtration, followed by thorough drying under reduced pressure in a desiccator. The prepared three-armed PCL was analyzed using gel permeation chromatography (GPC) and ^1^H NMR (AVANCE Neo 400, Bruker). ^1^H NMR spectroscopy was performed in CDCl_3_. The molecular weight was determined by GPC (two Tosoh columns TSKgel G3000H_HR_ and G5000H_HR_, JASCO system consisting of a UV-2075Plus intelligent UV/vis detector, PU-2080Plus intelligent HPLC pump, RI-2031Plus intelligent RI detector, and CO-2060Plus column oven [JASCO, Tokyo, Japan]). GPC measurements were performed at 45 °C using dimethylformamide (DMF) containing 10 mmol/L LiCl as the eluent. Poly(ethylene glycol)s with polydispersity indexes (PDIs) < 1.01 were used to generate the calibration curve.

### Degradable and Transformable Particle Preparation

The polymer solution was prepared by dissolving a known amount of PMDO-*co*-PEG and three-armed PCL (PCL/PMDO-*co*-PEG: 0, 0.5, 1.0, 3.0, 5.0, total polymer concentration: 20 mg/mL) in THF. The polymer solution in a 5 mL disposable polypropylene syringe (Terumo Co., Ltd., Tokyo, Japan) equipped with a 25 G (0.50 mm) needle (Terumo) was dropped into 25 mL of distilled water at a rate of 0.1 mL/min using a syringe pump (Precidor 5003, INFORS HT, Switzerland) with stirring at 200 rpm. A dialysis membrane (Spectra/Por 3 regenerated cellulose membrane tubing) with a molecular weight cutoff of 50,000 was then used to dialyze samples against water for 3 days, with the water changed every 4 h for the first 12 h, followed by daily intervals. After centrifugation at 7500 rpm (Model 5922 Kubota, Tokyo, Japan) at 5 °C, the supernatant was removed, and the precipitate was freeze-dried to obtain powdery particles.

### Degradation Test of the Prepared Particles

Degradation tests of the prepared particles were performed in aqueous NaOH. (1.0 mol/L) at 37 °C for 24 h (particle concentration of 1.0 wt %) as an acceleration test. After hydrolysis for a predetermined time, the sample solutions were neutralized, and the oligomers were recovered by lyophilization. The molecular weights of the hydrolyzed samples were determined by GPC using dimethylformamide (DMF).

### Preparation of Rod-Shaped Particles by Uniaxial Extension

The particles were dispersed in water (8 mL of 0.5 wt %) and added to 12 mL of 10 wt % PVA aqueous solution, and the mixture was gently stirred for 1 h in an ice bath. The dispersion was spread uniformly on a flat glass Petri dish and dried for 3 days to prepare a particle-dispersed PVA film. The prepared PVA film was cut into pieces of 1 × 6 cm, and both ends were clipped so that the uniaxially stretched portion was 3 cm. Subsequently, the PVA film was uniaxially stretched to 9 cm with heating at 60 °C. Approximately 4 cm of the central part of the stretched film was finely cut into pieces and immersed in distilled water at 5 °C for 1 week to dissolve the PVA. This solution was purified thrice by centrifugation at 5 °C and 7500 rpm for 30 min and then freeze-dried to obtain a powder sample. The aspect ratio (A.R.) of the rod-shaped particles was determined by dividing the major axis by the minor axis of the particles.

### Effect of the Particle Shape on Cellular Uptake in RAW 264.7 Cells

The effect of the particle shape on cellular uptake was determined by culturing RAW 264.7, in the presence of Nile Red-loaded spherical or rod-shaped particles and counting the number of uptakes. RAW 264.7 was seeded at a cell concentration of 2.0 × 10^4^ cells/cm^2^ and incubated at 37 °C for 24 h in FBS-containing DMEM. After removing the medium and washing with sterilized PBS, 900 μL of FBS-containing DMEM was added. PBS (100 μL) containing the sample particles was then added at a concentration of 1 mg/mL (final particle concentration of 100 μg/mL) and incubated at 37 °C for 24 h. The medium was removed, 10% (v/v) formalin was added, and the mixture was left to stand for 10 min for cell fixation. The number of particles taken up by RAW 264.7 cells was counted using a fluorescence microscope (BZ7000, Keyence, Osaka, Japan).

## Results and Discussion

### Preparation and Characterization of PMDO-*co*-PEG and Three-Armed PCL

**Scheme 1:**
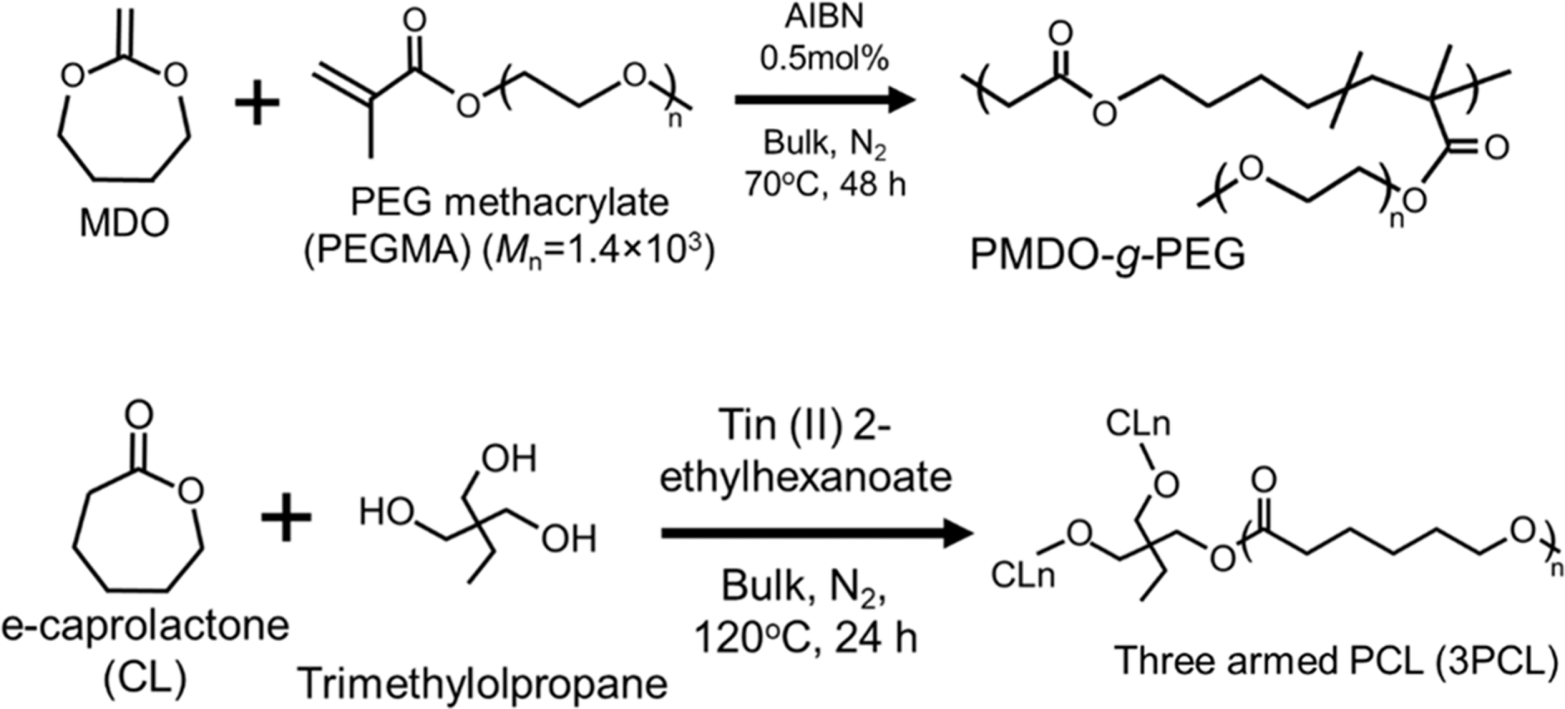
Preparation of Poly(2-methylene-1,3-dioxepane)-*co*-poly(ethylene glycol) PMDO-*co*-PEG and Three-Armed Poly(ε-caprolactone) (PCL)

PMDO-*co*-PEGs were synthesized by radical copolymerization of MDO and PEG (MDO:PEG = 100:1 and 200:1 in feed molar ratio) according to previous work^38,39^ (Scheme 1 upper part). The characteristics of PMDO-*co*-PEGs are summarized in Table 1. The ^1^H NMR spectrum of PMDO-*co*-PEG showed typical MDO (4.0–4.2 ppm) and PEG (3.6–3.8 ppm) peaks (Figure S1a). The PEG content in the copolymer was controlled by varying the PEG feed ratio. On the other hand, the conversion and yield were low due to the low reactivity of MDO.^38,39^

**Table 1:** Poly(2-methylene-1,3-dioxepane)-*co*-poly(ethylene glycol) Characterization

run	feed molar ratio MDO:PEG	*M*_n_^a^	*M*_w_/*M*_n_^a^	PEG content (mol %)^b^	conv. (%)^b^	yield (%)^c^
1	100:1	1.3 × 10^4^	2.1	0.95	24.2	22.2
2	200:1	1.9 × 10^4^	1.7	0.34	26.7	24.9

a Prepared by bulk polymerization using AIBN as the initiator at 65 °C for 24 h.

b Determined by GPC using poly(ethylene glycol) (PEG) as a molecular weight standard.

c Determined by ^1^H NMR in CDCl_3_ containing tetramethylsilane.

Three-armed PCL was prepared using TMP as an initiator (Scheme 1 lower part). Table 2 summarizes the results of the polymerization of three arms of PCL (3PCLs). The degree of polymerization of CL for one OH group (DP_CL_) was calculated from the integrated intensity ratio of the methylene group next to the oxygen atom in the main chain of PCL to the peak derived from the methyl group of the initiator TMP (Table 2 and Figure S1b). The DP_CL_s increased with an increasing molar ratio of the feed CL. In addition, the molecular weight of three-armed PCL increased with an increase in the feed molar ratio of CL. These results correspond to those of DP_CL_ calculated by ^1^H NMR, confirming that DP_CL_ can be controlled by the feed molar ratio of CL to TMP.

**Table 2:** Characterization of Three-Armed Poly(ε-caprolactone) (PCL)

sample		[CL]_0_/[–OH]_0_^a^	DP_CL_^b^	*M*_n_^c^	*M*_w_/*M*_n_^c^	yield (%)
1	3PCL-5	5.0	4.3	1.3 × 10^3^	1.5	90.4
2	3PCL-10	10.0	9.7	2.7 × 10^3^	1.6	93.5
3	3PCL-15	15.0	14.8	7.2 × 10^3^	1.1	93.6

a Molar ratio of the CL monomer to the initiating hydroxy group.

b Average degree of polymerization of CL arm segments of 3PCLs determined by ^1^H nuclear magnetic resonance NMR (CDCl_3_).

c Determined by gel permeation chromatography (GPC), poly(ethylene glycol) (PEG) standard.

**Figure 2:**
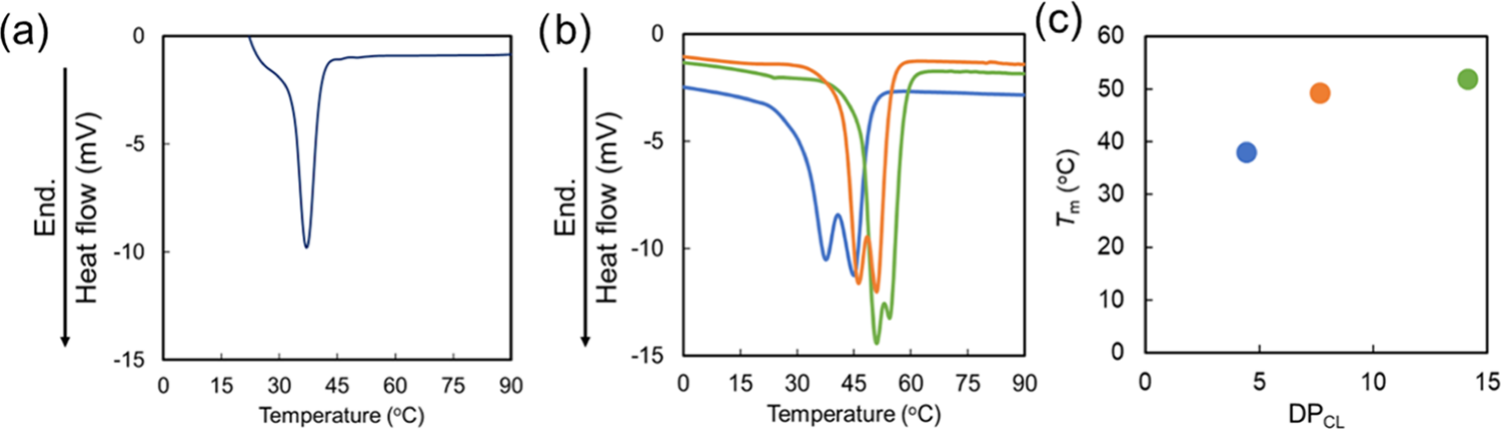
Differential scanning calorimetry (DSC) thermograms of (a) PMDO-*co*-PEG and (b) three-arm poly(ε-caprolactone) (3PCLs) during the heating scan (heating rate: 10 °C/min, 2nd heating run). Blue line: 3PCL-5, orange line: 3PCL-10, and green line: 3PCL-15. (c) Melting points of 3PCLs. Blue plot: 3PCL-5, orange plot: 3PCL-10, and green plot: 3PCL-15.

PMDO-*co*-PEG and all 3PCLs samples were examined to determine their *T*_m_ values using differential scanning calorimetry (DSC) as shown in Figure 2a,b. Samples were initially allowed to heat from −90 to 120 °C at 10 °C/min (1st run) with cooling to −90 °C. The temperature was then raised from −90 to 120 °C at 10 °C/min (2nd run). According to the DSC thermograms, the peak on the low-temperature side was defined as the melting point. In Figure 2a, the melting temperature (*T*_m_) of PMDO-*co*-PEG was around 39 °C. The melting point of 3PCLs increased in the range of 37.8–51.7 °C as DP_CL_ increased (Figures 2b,c and S2). This is attributable to the crystallinity of PCL that increases with increasing molecular weight. Compared to the melting point of linear PCL, which is usually observed around 60 °C, 3PCLs exhibited lower melting points because of their branched structure and relatively low molecular weights. Thus, we were able to have a melting point near the body temperature. The endothermic peak was broad, and two peaks were observed for all of the samples of 3PCLs. Figure S3 shows the DSC thermograms of the cooling and heating processes of 3PCL-5. The heating process yielded two broad peaks, whereas the cooling process yielded one peak. These results are similar to those reported in previous studies.^40,41^ These two peaks may be due to the formation of cyclic byproducts through the backbiting reaction of the terminal OH group to the ester group^41^ in the ring-opening polymerization of CL without terminal control. Therefore, the endothermic peak broadened owing to the contamination of cyclic byproducts and changes in the crystalline structure. In Table 2, broadened *M*_w_/*M*_n_ of 3PCL-5 and -10 may be affected by formation of cyclic byproducts. In addition, the broadening of the endothermic peak may have caused recrystallization during DSC measurements.^40,41^ In general, in the DSC measurements of crystalline polymers, a single endothermic peak that occurs when the crystal melts can be confirmed. However, measurements at relatively slow heating rates such as 10–20 °C/min allow sufficient time for the polymer in the melted state to reorganize into the original crystals. Therefore, the melting behavior has an exothermic peak due to recrystallization and an endothermic peak due to the melting of the recrystallized polymer. As a result, the melting behavior is a combination of the melting of the original crystal, heat of recrystallization, and melting of the recrystallized polymer.^40,41^ Therefore, two endothermic peaks are observed. In subsequent experiments, 3PCL-5 was used, which had a *T*_m_ of 37.8 °C, which is close to the body temperature.

### Degradable Particle Preparation and Characterization

**Figure 3:**
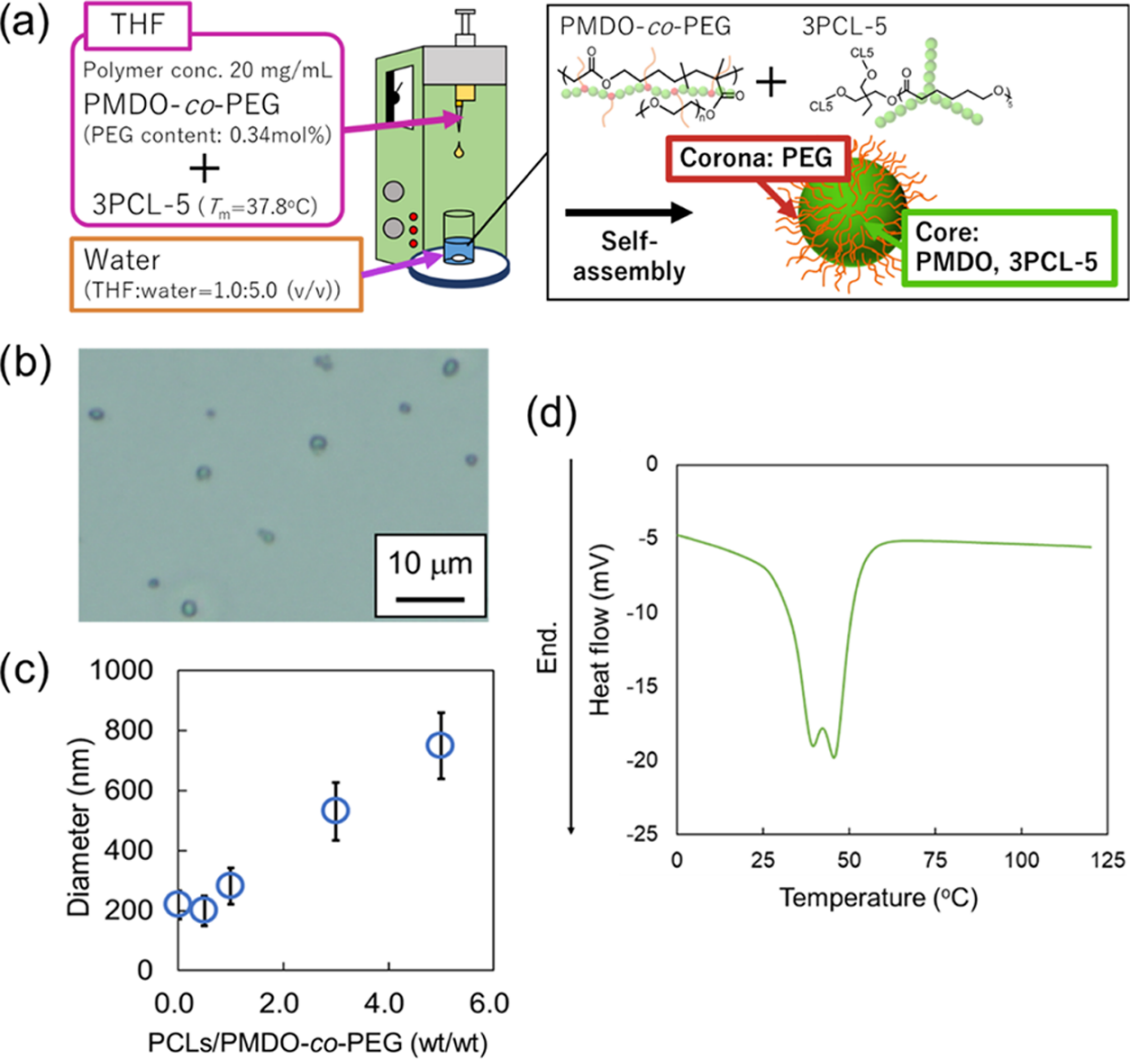
Preparation and characterization of spherical particles. (a) Preparation method of particles. (b) Microscopic view of the prepared particles (3PCL-5/PMDO-*co*-PEG: 5.0). (c) Diameter of particles made with various compositions as determined by dynamic light scattering (DLS). Data are presented as mean ± standard deviation (SD). (d) Differential scanning calorimetry (DSC) thermograms of the prepared particles composed of three-arm poly(ε-caprolactone) (3PCL-5) and PMDO-*co*-PEG (3PCL-5/PMDO-*co*-PEG: 5.0) during the heating scan (heating rate: 10 °C/min, 2nd heating run).

The particles were prepared according to previous reports.^33,34^ THF solutions of PMDO-*co*-PEG and 3PCL-5 were added dropwise to ultrapure water using a syringe pump, while the water was gently stirred (Figure 3a). Optical microscopy revealed that the particles formed were spherical (Figure 3b, PCL/PMDO-*co*-PEG: 5.0). Alone, 3PCL-5 did not form particles but formed aggregates in water because no hydrophilic segments were present. On the other hand, the particles were formed by mixing PMDO-*co*-PEG and 3PCL-5. This may be due to the PEG introduced into PMDO-*co*-PEG being exposed on the particle surface. By increasing the PCL weight ratio in total polymer weight, the average diameter of the prepared particles increased from 200 nm without 3PCL-5 to 800 nm with a 5.0 times higher amount of 3PCL-5 than the copolymer (Figures 3c and S3). This is due to the incorporation of 3PCL-5 into the core, which increases the particle size. Therefore, the core of the self-assembled particles is composed of a mixture of PMDO-*co*-PEG and 3PCL-5 with PEG in the corona layer. Thus, the dispersibility of the prepared particles was sufficiently high because of the effect of PEG in the corona layer for at least 1 week in water. In addition, the DSC thermograms of P(MDO-*co*-PEG) and particles are shown in Figures 2a and 3d, respectively. The single peak of the melting point of P(MDO-*co*-PEG) was shown at 39 °C (Figure 2a). On the other hand, the melting point of the prepared particles was 39 °C (the peak on the low-temperature side) slightly increased compared with the melting point of 3PCL-5 (37.8 °C). It was suggested that the melting point of particles mixed PMDO-*co*-PEG with 3PCL-5 slightly increases.

**Figure 4:**
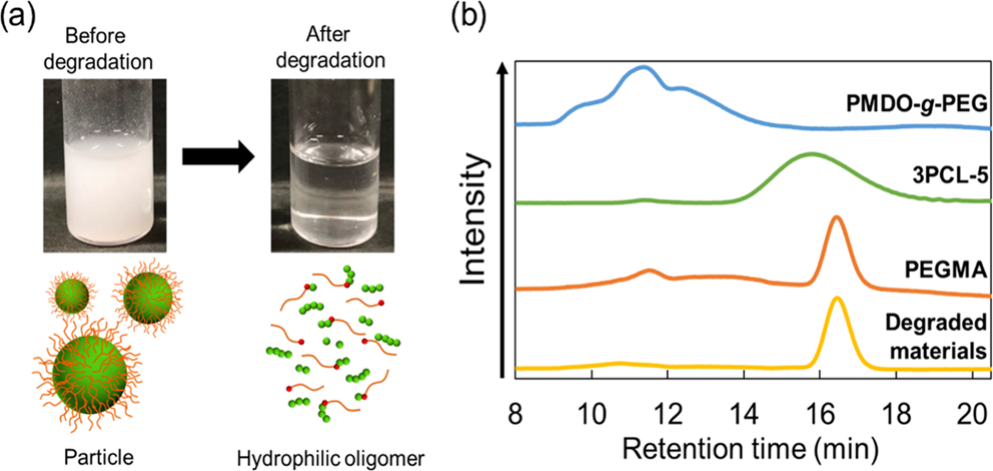
Degradation test of the prepared degradable particles. (a) Visible appearances of poly(2-methylene-1,3-dioxepane)-*co*-poly(ethylene glycol) P(MDO-*co*-PEG) and three-arm poly(ε-caprolactone) (3PCL-5) particle dispersion before and after degradation under alkaline conditions (1.5 mol/L NaOH aq). (b) Gel permeation chromatography (GPC) charts of the polymers and degraded materials. Blue line: PMDO-*ran*-PEG, green line: three-arm poly(ε-caprolactone) (3PCL-5), orange line: poly(ethylene glycol) monomethacrylate (PEG), and yellow line: degraded materials.

An accelerated hydrolysis test was performed in 1.5 mol/L NaOH aqueous solution at 37 °C for 1 d. As shown in Figure 4a, the particle dispersion became transparent following degradation. This is attributable to the ester groups of PMDO-*co*-PEG and 3PCL-5 that hydrolyzed and became water-soluble. The molecular weights of the degradants were evaluated by GPC measurements, and the results are listed in Figure 4b. The GPC peak derived from PMDO-*co*-PEG completely disappeared, and the peaks derived from 3PCL-5 nearly diminished after hydrolysis. A new peak appeared at 1.8 × 10^3^ for the degraded materials, which corresponded to the GPC peak derived from PEG. From this result, the molecular weight decreased to the same level as that of the raw materials PMDO-*co*-PEG and 3PCL-5 after degradation.

### Preparation of Rod-Shaped Particles by Uniaxial Extension

**Figure 5:**
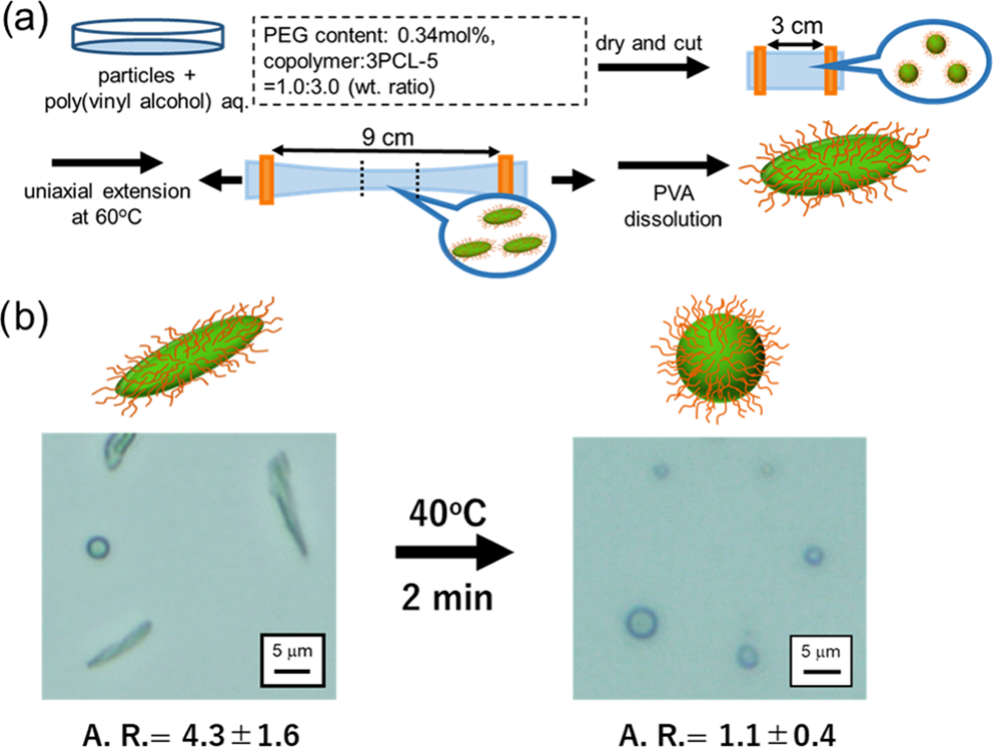
Preparation of transformable rod-shaped particles via uniaxial extension. (a) Schematic illustration of preparation of rod-shaped particles. (b) Microscopic observation of rod-shaped particles (left) and spherical shape particles (right) after exceeding the *T*_m_ of three-arm poly(ε-caprolactone) (3PCL-5, 3PCL-5/PMDO-*co*-PEG: 5.0) dispersed in water. The aspect ratio (A.R.) of rod and spherical particles is indicated under the microscopic images (*n* = 30).

Mitragotri et al. reported that rod-shaped particles were prepared by uniaxial stretching of a PVA film in which the particles were dispersed.^16−18^ Here, rod-shaped particles were prepared by using the same method. A PVA film containing particles of PMDO-*co*-PEG:3PCL-5 = 1:3 (wt. ratio) and PEG content in PMDO-*co*-PEG of 0.34 mol % was uniaxially extended three times its original length with continuous film heating (Figure 5a). The center part of the extended films was immersed in cold water with stirring at 5 °C for 3 days to remove PVA, and rod-shaped particles were obtained. Figure 5b (left) indicates the formation of rod-shaped particles. The calculated A.R. of the rod-shaped particles was 4.3 ± 1.6 (*n* = 30). After uniaxial stretching, the rod shape is maintained. At 40 °C near the melting point of the core, the rod-shaped particles quickly returned to spherical shapes with an A.R. value of 1.1 ± 0.4 in 2 min (Figure 5b, right). However, 25 °C below the melting point, the shape of the rod-shaped particles remained unchanged. Therefore, the melting point of 3PCL-5 affects the deformation of the particles from rod to sphere, and the shape of the particles is mediated by the temperature.

### Effect of the Particle Shape on Cellular Uptake in RAW 264.7 Cells

**Figure 6:**
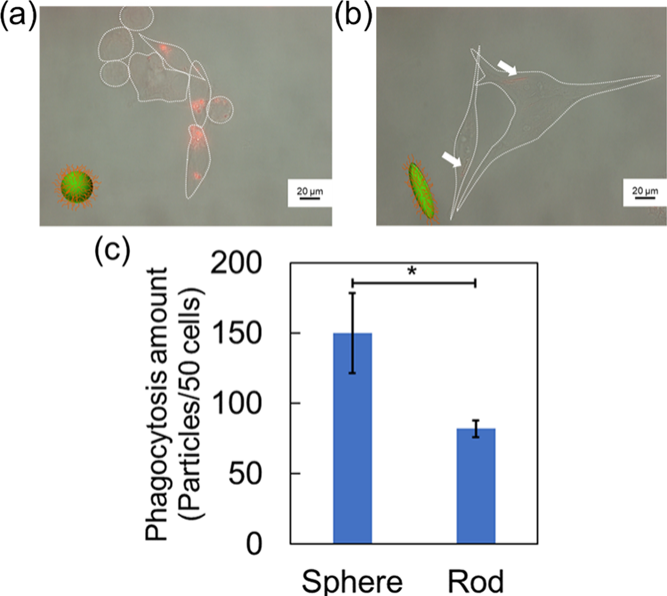
Cellular uptake of spherical and rod-shaped particles. (a) Microscopic image of macrophage-phagocytized spherical particles. The scale bar represents 20 μm. (b) Microscopic image of macrophage-phagocytized rod-shaped particles. The scale bar represents 20 μm. (c) Phagocytosis amount of spherical and rod-shaped particles. Data are expressed as the mean with SD (*n* = 3). The asterisk indicates significant differences at *p* < 0.05.

The cytotoxic activity of the prepared particles was assessed with RAW 264.7 macrophage cell cultures using a live/dead assay. Figure S4 showcases the particle concentration-dependent changes in the cell viability. More than 95% of the cells were viable in the presence of spherical particles up to a particle concentration of 100 μg/mL, suggesting that the polymer particles were nontoxic at this concentration range. Spherical or rod-shaped particles were dispersed in PBS solution, added to RAW 264.7 cell cultures at a final particle concentration of 100 μg/mL, and incubated for 24 h. Nile Red-encapsulated particles were used in this assay. The rod-shaped particles used in the cellular uptake are not transformable particles for spherical particles at 37 °C because the melting point of the prepared particles was around 39 °C (Figure 2b). After incubation, the number of phagocytosed spherical or rod-shaped particles was determined using a fluorescence microscope (Figure 6). Both spherical and rod-shaped particles were observed within the macrophages (Figures 6a,b, and S5). From the fluorescence microscopic images, we see that the spherical particles were clearly absorbed by the cells compared to the rod-shaped particles. The number of particles phagocytized by macrophages per 50 cells was calculated using fluorescence microscopy (Figure 6c), which revealed that significantly more amounts of spherical particles were phagocytized, compared to rod-shaped particles. This result agrees with previous work regarding the cellular uptake of rod-shaped particles.^16−18^ Phagocytosis of rod-shaped particles was suppressed because the contact angle of rod-shaped particles with macrophages affected actin remodeling.^17^ At less than 45° of contact angle between the particle major axis and the cells, actin filament elongation and cellular uptake occurred. On the other hand, above 45°, actin filaments are insufficiently elongated and cell uptake does not occur. In this study, the cellular uptake of the prepared rod-shaped particles occurred in a similar fashion as in the previous reports. Therefore, it is considered that the phagocytosis amounts of particles differed depending on the particle shape.

In addition, the result of cellular uptake of spherical particles that were obtained from rod-shaped particles by heating at 40 °C for 24 h is shown in Figure S6. Red fluorescence was derived from Nile Red in spherical-shaped particles. The result indicated that the spherical particles after shape change from the rod-shaped one were uptaken to cells similarly to the spherical particles as prepared. This indicated the minimal influence of the PVA if it remained on the particle surfaces. Therefore, the amount of particle phagocytosis changed depending on the particle shape. In addition, reverting from a rod shape to a spherical shape is expected to increase the cellular uptake. The prepared particles are expected to be applied as degradable DDS carriers that enable control of the interaction with cells by controlling the particle shape by temperature change using external stimuli such as focused sonophoresis and/or alternating magnetic fields.

## Conclusions

In this study, biodegradable and transformable particles composed of PMDO-*co*-PEG and 3PCL-5 were prepared. The melting point of the prepared particles was controlled by the molecular weight of the three-armed PCLs. After degradation, the molecular weight of the degradants decreased to approximately 2000 g/mol, which was identical to the molecular weight of PEG. These results indicated that the ester groups of PMDO-*co*-PEG and 3PCL-5 were completely degraded. Rod-shaped particles were prepared by uniaxial stretching of a PVA film containing spherical particles above the melting point of 3PCL-5. The prepared particles showed a shape change from rod to spherical with increasing temperature. The phagocytic uptake amounts of spherical particles were higher than those of rod-shaped particles, further suggesting that the phagocytic uptake can be regulated by the particle shape. These results strongly suggest that the prepared particles can be applied as degradable DDS carriers that enable control of the interaction with cells via particle shape induced by changes in temperature.

## Supporting Information Available

^1^H NMR spectra of PMDO-*g*-PEG and PCL (3PCL-5) in CDCl_3_, analysis data of the synthesized polymer (PMDO-*g*-PEG and 3PCL-5) by ^1^H NMR, DSC thermograms of 3PCL-5, -10, and -15 during the cooling scan and the heating scan (heating and cooling rate: 10 °C/min), analysis of melting point change depending on the molecular weight of the synthesized PCL by DSC (heating and cooling processes), diameter of particles made with various compositions as determined by dynamic light scattering (DLS), cytotoxic activity of the prepared particles assessed in RAW 264.7 macrophage cells, and optical and fluorescence microscopic images of the macrophages incubated with either spherical or rod-shaped particles encapsulated with Nile Red (PDF)
