# Supplementary material for: Preparation of Degradable and Transformable Core–Corona-Type Particles that Control Cellular Uptake by Thermal Shape Change
Source: ACS Biomater Sci Eng. 2024 Jan 20;10(2):897–904. doi: 10.1021/acsbiomaterials.3c01554 (PMC10865287; doi:10.1021/acsbiomaterials.3c01554)
Supplement: Supplementary file 1 — ab3c01554_si_001.pdf [file ab3c01554_si_001.pdf]

Supporting Information

Preparation of degradable and transformable core-corona type particles that control cellular uptake by thermal shape change

Syuuhei Komatsu [1], Satoshi Yamada[1], Akihiko Kikuchi [1]*

[1] Department of Materials Science and Technology, Tokyo University of Science, 6-3-1 Niijuku, Katsushika, Tokyo 125-8585, Japan

* Corresponding author: Akihiko Kikuchi

Phone: +81-3-5876-1415; Fax: +81-3-5876-1639; E-mail: kikuchia@rs.tus.ac.jp

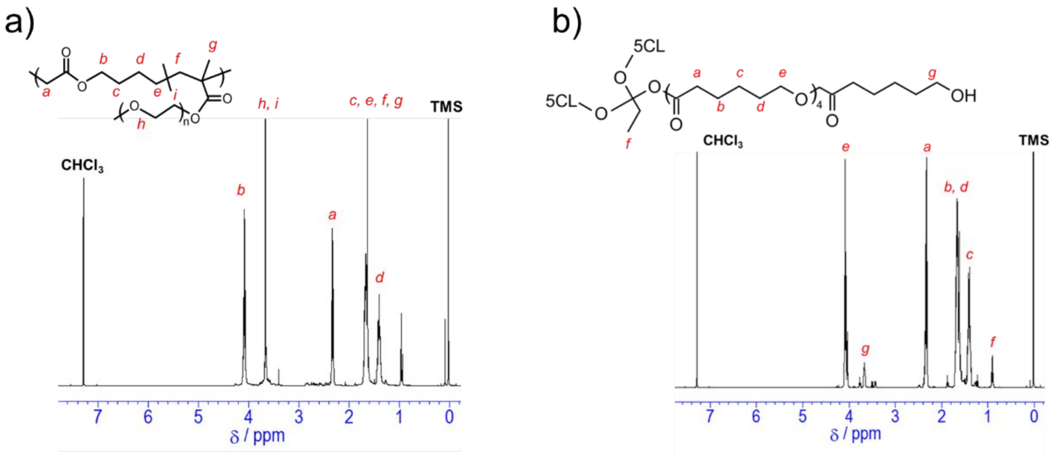

Figure S1 ^{1}H NMR spectra of a) PMDO-*g*-PEG and b) PCL (3PCL-5) in $CDCl_3$.

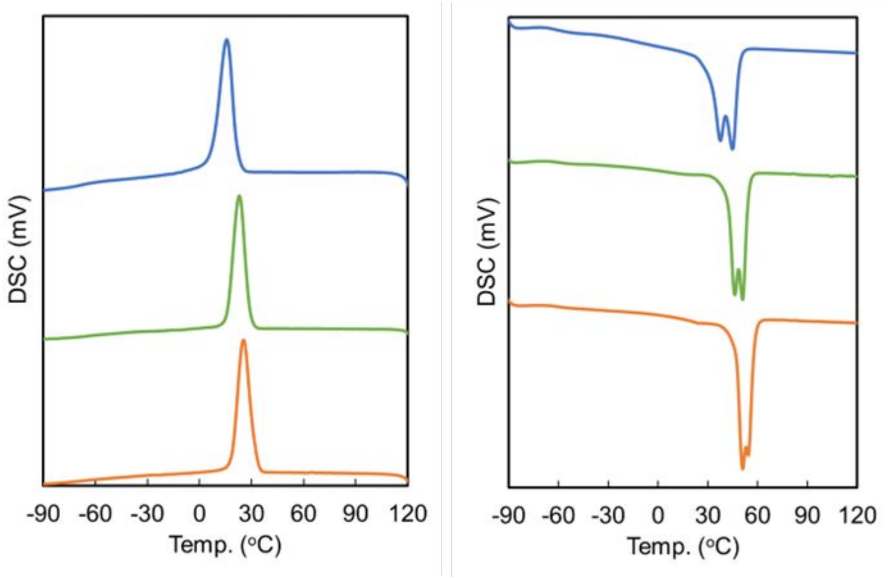

Figure S2 DSC thermograms of 3PCL-5 (blue line),10 (green line),15 (orange line) during the cooling scan (left) and the heating scan (right) (heating and cooling rate: 10°C/min).

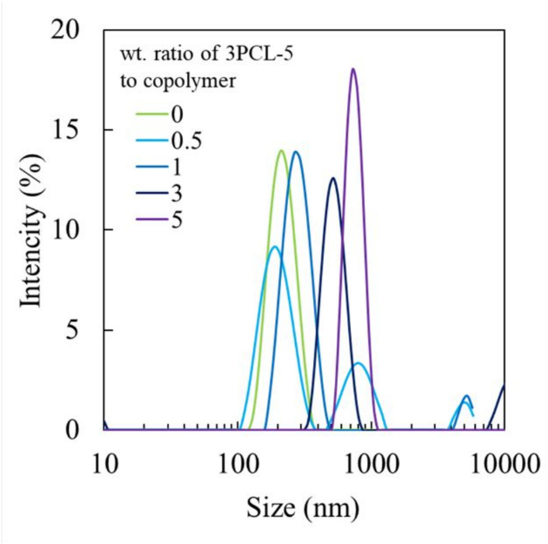

Figure S3 Diameter of particles made with various compositions as determined by dynamic light scattering (DLS).

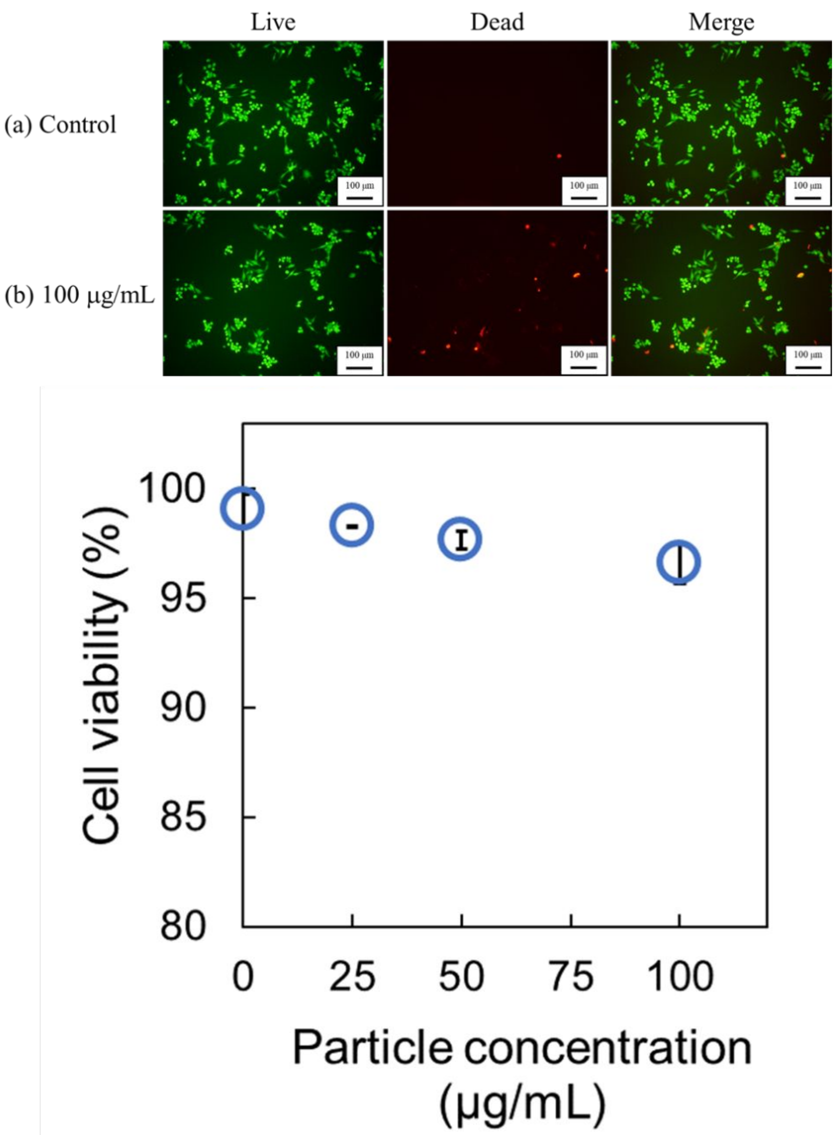

Figure S4 The cytotoxic activity of prepared particles was assessed in RAW 264.7 macrophage cells.

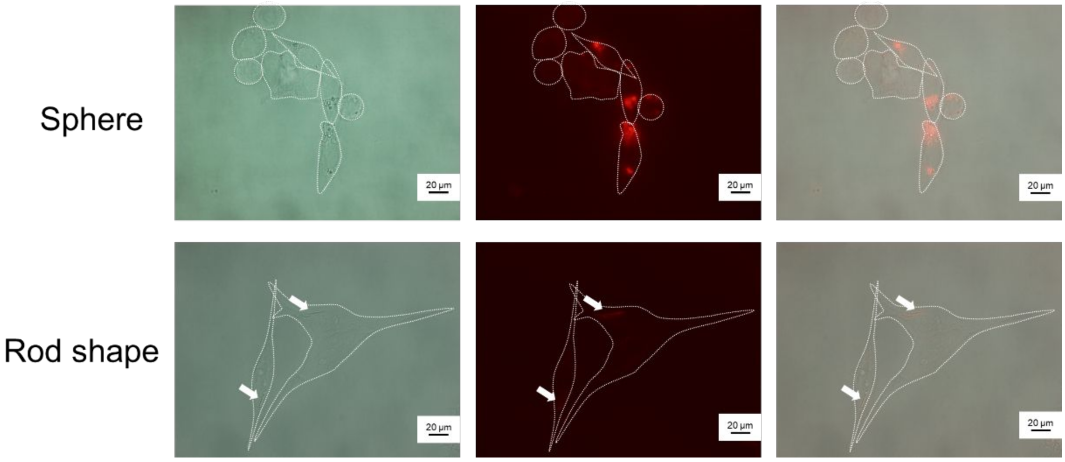

Figure S5 Optical and fluorescent microscopic images of the macrophages incubated with either spherical or rod-shaped particles encapsulated with nile red. Left: optical image of the macrophage cultures, middle: fluorescent images derived from nile red fluorescence, right: merged images.

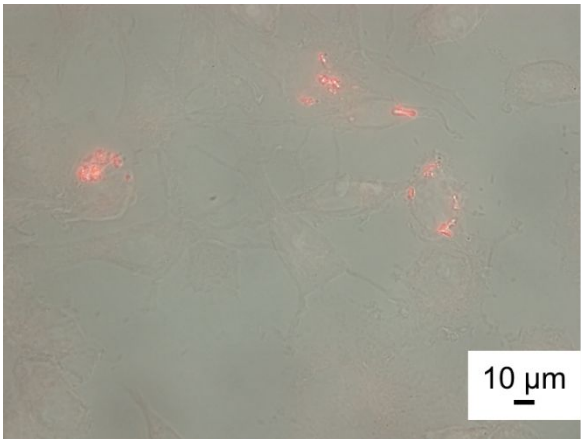

Figure S6 Optical and fluorescent microscopic images of the macrophages incubated with spherical-shaped particles after shape change from rod to sphere by incubating above the core T_m.
